# Spontaneous periduodenal hematoma: a rare surgical and radiological conundrum

**DOI:** 10.1093/jscr/rjad133

**Published:** 2023-03-14

**Authors:** Maria Padar, Amie Rieseberg, Rasika Hendahewa

**Affiliations:** Surgical Department, Caboolture Hospital, Caboolture, QLD Australia; Radiology Department, Caboolture Hospital, Caboolture, QLD, Australia; Surgical Department, Caboolture Hospital, Caboolture, QLD Australia

**Keywords:** Periduodenal hematoma, nontraumatic hematoma, duodenum hematoma, unknown cause, conservative management, radiological findings

## Abstract

This is a case of a 68-year-old female with spontaneous and rare periduodenal hematoma of unknown origin without any signs or symptoms of duodenal stenosis. All causes of known precipitating factors, such as trauma, intervention, anticoagulation, pancreatitis or malignant processes, were ruled out. She was managed conservatively, with a series of radiological investigations performed throughout her 7-day admission to further define stability and investigate the possible cause for the periduodenal hemorrhage. A repeat magnetic resonance imaging 4 weeks later showed near resolution of the hematoma. The underlying cause in this case remains unclear.

## INTRODUCTION

Periduodenal hematoma without precipitating trauma is a rare entity. Trauma, including various intervention-related injuries of the duodenum causing intramural or periduodenal hematoma has been presented multiple times in the literature. Spontaneous intramural hematoma is reported more frequently as a complication of anticoagulation therapy since its introduction and wide usage. Another common trigger is pancreatitis. There are, however, other causes, including congenital, inflammatory, vascular or ischemic often with a presenting symptom of duodenal stenosis.

## CASE REPORT

A 68-year-old female presented to the emergency department with a 3-day history of severe epigastric pain radiating to the back and the right shoulder with associated nausea, without vomiting. She had no preceding trauma. Previous surgeries of total abdominal hysterectomy, bilateral oophorectomy were confined to the lower abdomen. Her only medical issue was hypertension, not well controlled despite four antihypertensive agents with high systolic blood pressure during her stay. She was also on aspirin.

She was hemodynamically stable with no fever. On her initial assessment, she had a soft abdomen with RUQ tenderness, no obvious palpable mass. Bloods were unremarkable including WCC: 10.3 10^9^/L, normal lipase, lactate, liver and renal function tests, except a new Hgb drop of 103 g/L (5 months ago documented 144 g/L). Coagulation profile was normal.

Since the initial working diagnosis was cholecystitis, she had an abdominal ultrasound examination, which showed a normal gallbladder and pancreas, and long-standing liver hemangiomas (compared with previous images). There was a small amount of nonspecific perinephric, subhepatic fluid.

**Figure 1:**
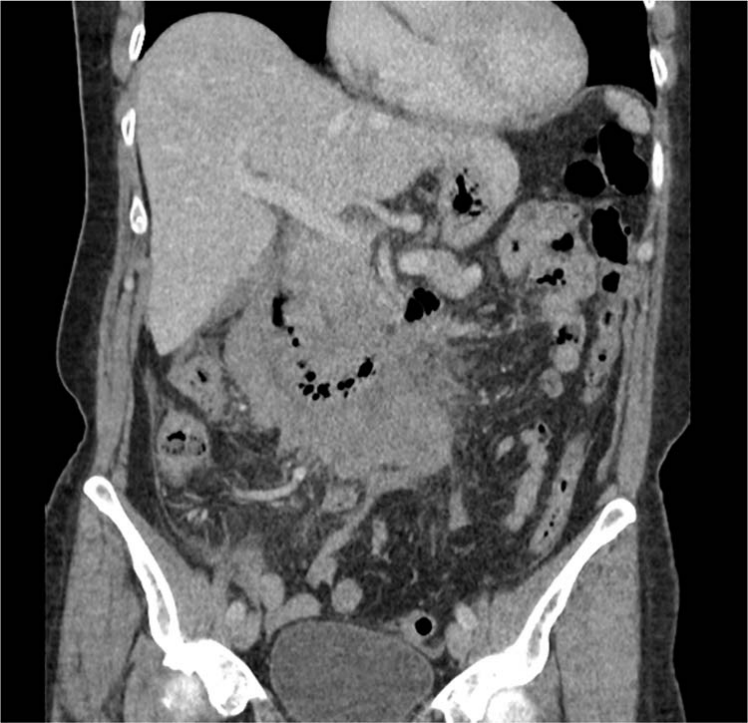
CT abdomen, coronal view. Hyperdense fluid centered around the duodenum and tracking into the paracolic gutters. Appearance concerning for paraduodenal hemorrhage, infiltrating mass was considered less likely.

**Figure 2:**
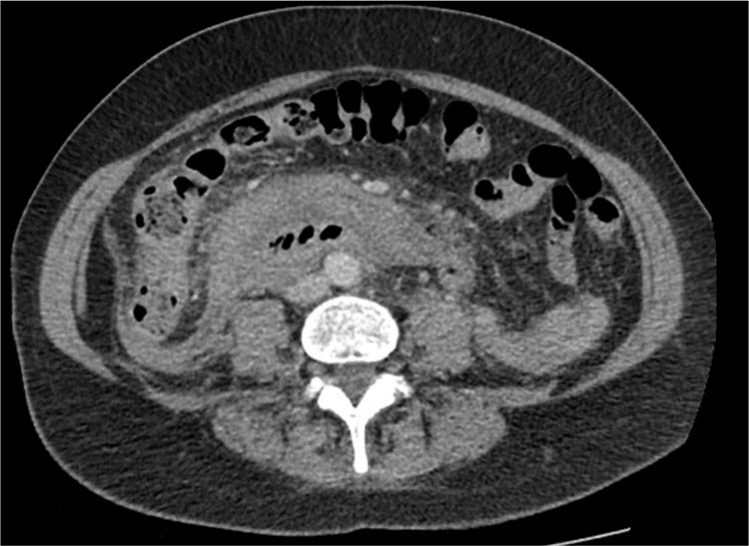
CT abdomen, axial view. Hyperdense fluid centered around the duodenum and tracking into the paracolic gutters. Appearance concerning for paraduodenal hemorrhage, infiltrating mass was considered less likely.

The radiologist suggested a computer tomography (CT) scan to further explore the potential causes for this fluid. The CT abdomen and pelvis revealed extensive oedema and induration surrounding the duodenum with a complex hyperdense fluid, extending from this site to the paracolic gutters and into the pelvis. While these findings are suspicious of a paraduodenal hemorrhage from a ruptured duodenal ulcer and hemoperitoneum, they were not typical for perforated duodenal ulcer or erosion into the blood vessels. Differentials were an infiltrating mass either fibrotic or of a lymphoproliferative origin. However, this could not explain the complex fluid in the abdomen (Figs 1 and 2).

**Figure 3:**
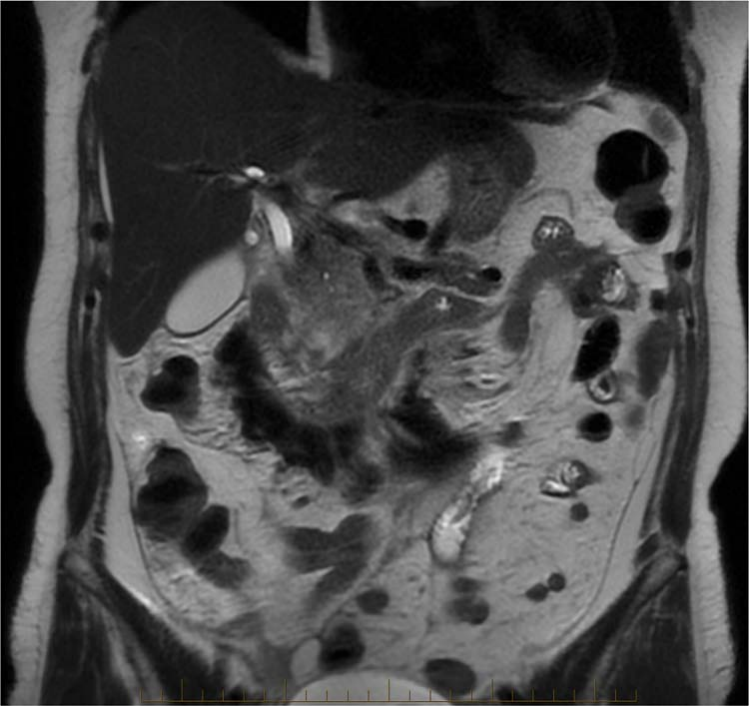
MRI abdomen, coronal view. T2 hypointense, T1 iso to hyperintense material surrounding D2/D3 without enhancement in keeping with hemorrhage. No vascular or bowel wall lesion identified.

**Figure 4:**
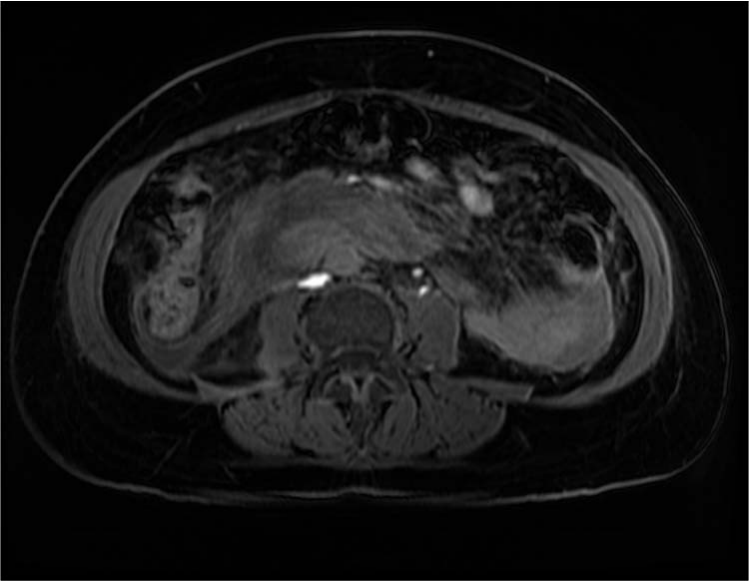
MRI abdomen, axial view. T2 hypointense, T1 iso to hyperintense material surrounding D2/D3 without enhancement in keeping with hemorrhage. No vascular or bowel wall lesion identified.

After further discussion with the reporting radiologist, a magnetic resonance imaging (MRI) was performed, which confirmed the presence of an acute periduodenal hematoma surrounding D2 and D3, lying in the retroperitoneal space, anterior to the right perirenal space. Etiology was uncertain. No abnormal enhancement within the bowel to suggest a bowel wall lesion or infiltrating mass (Figs 3 and 4).

She was admitted under the surgical team for observation. Patient kept nil by mouth, IV fluids were given, as well as IV pantoprazole. She had ongoing pain, worsening with movement, and therefore, the pain management team was involved. There was a significant drop of Hgb from 103 to 88 g/L the next day, without hemodynamical instability, hence an urgent CT angiography was requested to rule out active bleeding.

**Figure 5:**
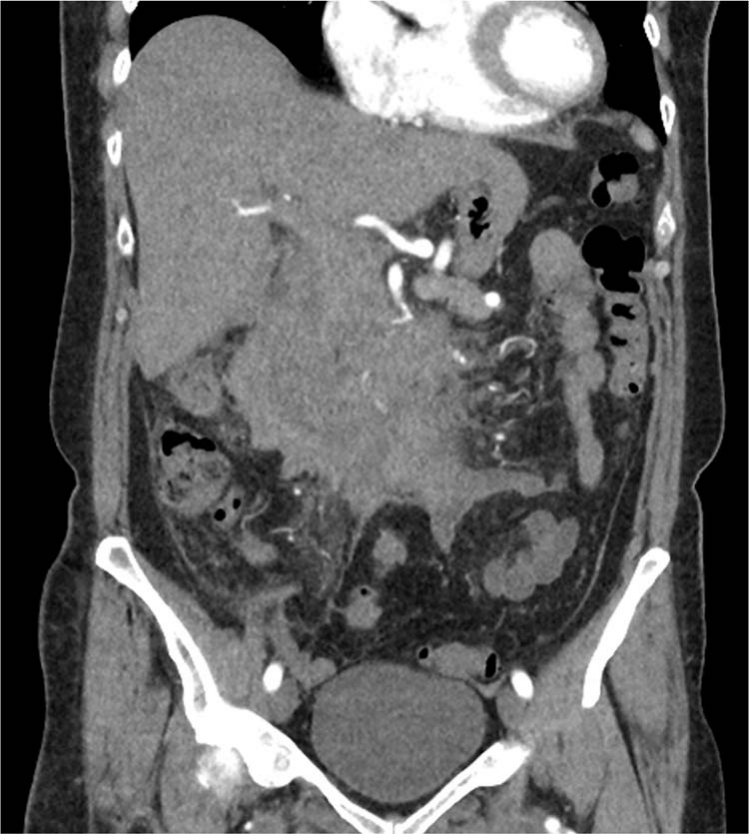
CT triple phase, coronal view. Stable periduodenal hematoma, no acute hemorrhage or vascular abnormality identified.

**Figure 6:**
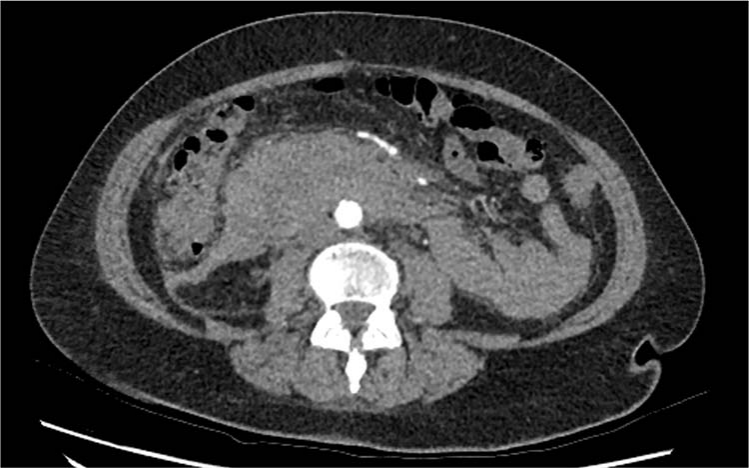
CT triple phase, axial view. Stable periduodenal hematoma, no acute hemorrhage or vascular abnormality identified.

It showed stabile appearance of the periduodenal collection surrounding D2 and D3 with retroperitoneal hemorrhage extending into the pelvis. Normal contrast opacification of the coeliac axis, SMA renal arteries and IMA (Figs 5 and 6).

**Figure 7:**
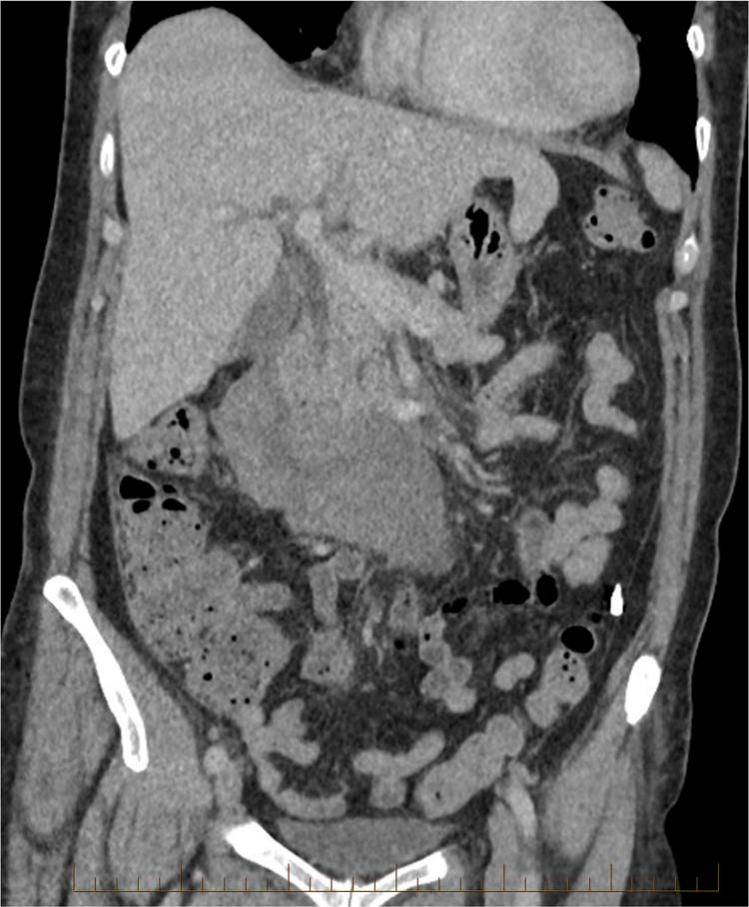
CT abdomen, coronal view. Slight interval decrease in size of previously identified periduodenal hematoma.

**Figure 8:**
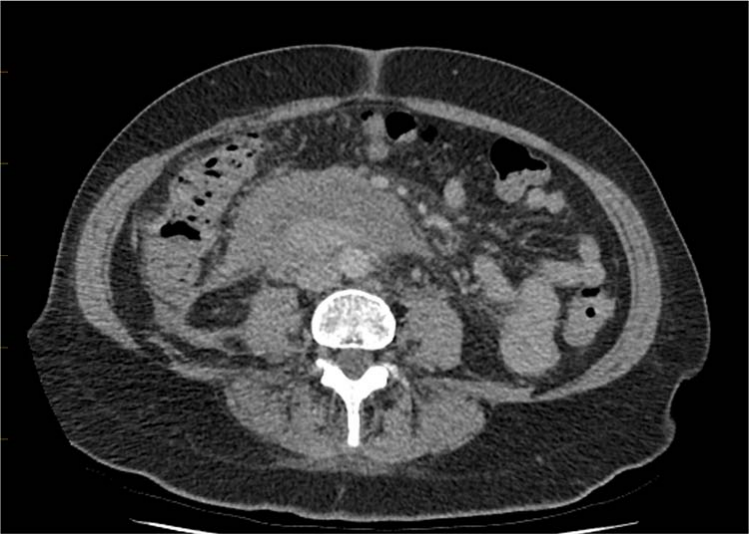
CT abdomen, axial view. Slight interval decrease in size of previously identified periduodenal hematoma.

Continued observation, analgesia and fasting. On Day 2 post-admission, the patient had an urgent upper gastrointestinal endoscopy, which showed no abnormality. No ulcer, compression or inflammation was found. After the gastroscopy, the patient was started on a normal diet, which she tolerated well, and her pain had also improved. A progress CT was performed on Day 5, which showed a mild increase of the periduodenal collection with mass effect on the duodenum and evidence of mild biliary tree dilatation with the common bile duct measuring 11 mm. There was no sign of gastric outlet obstruction (Figs 7 and 8).

The patient recovered well and was discharged on Day 7 with a stable Hgb of 110.

**Figure 9:**
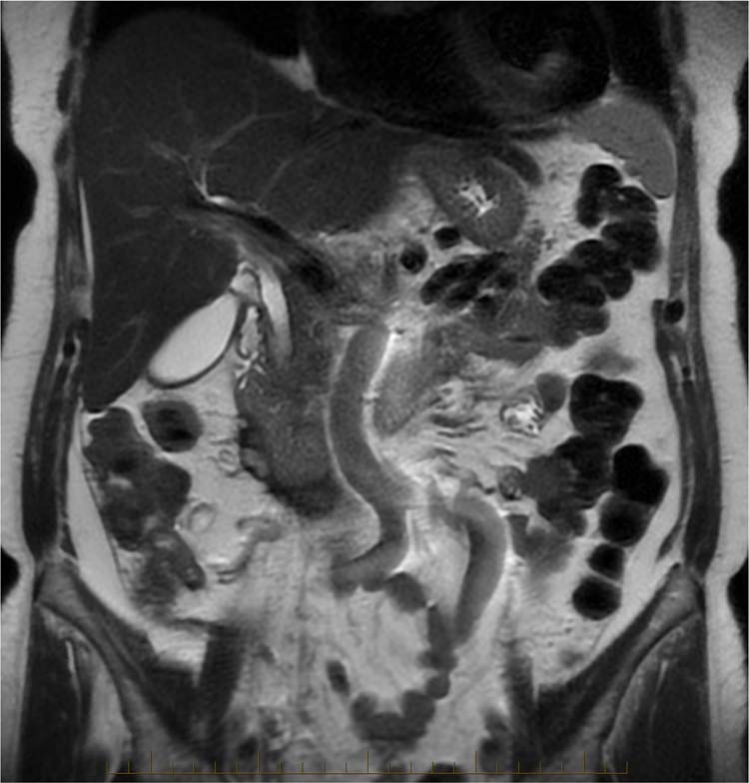
MRI abdomen, coronal view. Resolving paraduodenal hematoma.

**Figure 10:**
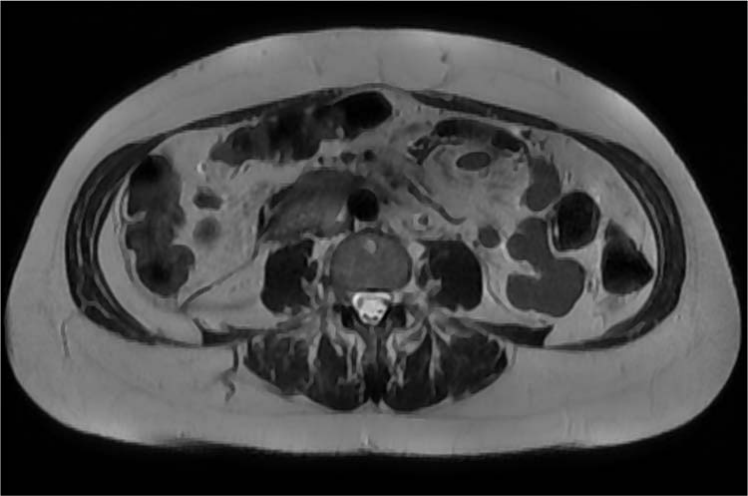
MRI abdomen, axial view. Resolving paraduodenal hematoma.

She was reviewed in the surgical outpatient clinic 4 weeks later. She was still experiencing mild epigastric, right upper quadrant pain. The repeat Hgb was 133. Progress MRI abdomen and pancreas showed resolving hematoma, slight prominence of D2 and D3 of the duodenal wall with no discrete mass seen. No evidence of duodenal obstruction, prominent CBD of 8 mm (Figs 9 and 10).

## DISCUSSION

The first autopsy confirmed intramural intestinal hematoma published by James McLauchlan [1]. In the early 1900s, there were several reported cases of intestinal hematoma, either caused by hemophilia and hemorrhagic purpura or reported trauma. The first preoperatively, radiologically described and diagnosed jejunal hematoma case was published by Kjell Liverud [3]. Benjamin Felson and Emanuel J. Levin [2] published four cases of radiologically confirmed duodenal hematoma. All patients had gastrointestinal series of oral contrast studies confirming the diagnosis, which was surgically proven in three cases [3]. With the advancement of radiological technics, including CT and MRI, early diagnosis and conservative management of suitable patients became more frequent. MRI diagnosis with a well-defined concentric ring configuration described in two duodenal hematoma cases, because of paramagnetic properties of iron within the hematoma, attributed to Peter F. Hahn *et al*. [4].

Duodenal hematoma can be intramural or periduodenal, frequently caused by trauma.

There are various causes of duodenal hematoma, such as trauma including intervention-related injuries; pancreatitis and its sequels; malignancy, vascular anomalies, with subsequent aneurysmal rupture; vasculitis, coagulopathy, duodenitis associated with Crohn’s disease, duodenal ulcer and perforation; congenital causes, for example: duplication cyst, duodenal diverticulum and its complications. Most of these cases are associated with duodenal stenosis [5].

The rare, nontraumatic intramural hematoma, however, can be associated with anticoagulation and coagulopathy causing duodenal obstruction [6, 7].

This case of periduodenal hematoma demonstrates a rare entity, which, with appropriate imaging investigation, can be confidently managed conservatively. The underlying cause in this case, however, remains unclear.

## CONFLICT OF INTEREST STATEMENT

None declared.

## FUNDING

None.

## DATA AVAILABILITY

The documents used during the current study are available from the corresponding author on reasonable request.

## INFORMED CONSENT

Informed consent was obtained from the patient for publication of this case report and accompanying figures.
